# Childhood anemia and household salt iodization in Peru: population disparities among children aged 6–59 months

**DOI:** 10.3389/fnut.2026.1865961

**Published:** 2026-06-12

**Authors:** Jhofree Einstein Briceño-Chavez, Lily Mabel Portal-Valqui, Adriana Mishell Yoplac-Oyarce, Juan Carlos Bustamante-Rodríguez, Jhosmer Ballena-Caicedo, Fiorella E. Zuzunaga-Montoya, Víctor Juan Vera-Ponce

**Affiliations:** Facultad de Medicina (FAMED), Universidad Nacional Toribio Rodríguez de Mendoza de Amazonas (UNTRM), Chachapoyas, Peru

**Keywords:** anemia, child nutrition, health disparities, iodine, iodized salt, Peru

## Abstract

**Background:**

Childhood anemia remains a priority public health problem in Peru. Although universal salt iodization is an established strategy to prevent iodine deficiency disorders, evidence on household-level salt iodization and disparities in anemia remains limited.

**Objective:**

To estimate anemia prevalence and evaluate disparities according to the adequacy of iodine content in household salt among girls and boys aged 6 to 59 months in Peru, as well as to explore variation according to area of residence and household wealth level.

**Methods:**

Analytical cross-sectional study using data from the Peruvian Demographic and Family Health Survey (ENDES) 2014–2023. We included 110,561 children aged 6 to 59 months with valid hemoglobin measurement and a household salt iodine rapid test. Anemia was defined as altitude-adjusted hemoglobin <11.0 g/dL. The main exposure was inadequate household salt iodization (<15 ppm), compared with adequate iodization (≥15 ppm). Weighted prevalences, absolute gaps, and crude and adjusted prevalence ratios (PRs) were estimated using Poisson regression for complex survey design, adjusting for age and sex.

**Results:**

The average weighted prevalence of anemia was 31.1% (95% CI: 30.7–31.6). Anemia was more frequent in households with inadequately iodized salt than in those with adequately iodized salt (35.73% vs. 30.49%). The absolute gap was 5.24 percentage points (95% CI: 3.7–6.7), and the crude PR was 1.17 (95% CI: 1.13–1.21). After adjustment for age and sex, the association remained (aPR = 1.16; 95% CI: 1.12–1.20). Absolute differences varied by area of residence and wealth quintile; no statistical evidence of additive-scale interaction was observed with extreme poverty or rural residence. Including 2024 as a sensitivity analysis did not materially change the estimate.

**Conclusion:**

Anemia was more frequent among children living in households with inadequately iodized salt. The findings support an interpretation centered on population disparities and nutritional vulnerability rather than on a direct causal relationship attributable to iodine in household salt.

## 1 Introduction

Childhood anemia is a priority public health problem worldwide. According to the Global Burden of Disease Study 2021, the estimated global prevalence was 41.4% among children younger than 5 years (1). The World Health Organization (WHO) estimates that around 40% of children aged 6–59 months are affected by anemia, with greater vulnerability among younger children and in low- and lower-middle-income settings (2). In Latin America and the Caribbean, a meta-analysis reported an overall anemia prevalence of 28.56% in children younger than 12 years and 32.93% in preschoolers (3), whereas in Peru the 2024 Demographic and Family Health Survey (ENDES) reported a prevalence of 33.7% among children aged 6–35 months (4). Beyond its high prevalence, anemia in children younger than five years is associated with increased morbidity and adverse effects on child development; in severe cases, it may compromise cognitive and motor development, with potential consequences across the life course (2, 5).

Although iron deficiency is the most common cause of anemia, this condition is multifactorial and may coexist with other micronutrient deficiencies that also contribute to its population burden (6). Among them, iodine is a micronutrient of interest because of its relationship with thyroid function; thyroid hormones participate in hematopoietic processes and could influence erythroid differentiation and hematologic parameters (7, 8). Consistently, observational studies have described associations between iodine insufficiency and lower hemoglobin levels. For example, among Nepalese schoolchildren, iodine insufficiency has been associated with lower hemoglobin concentrations and a higher frequency of anemia (9). In contrast, among pregnant women, lower hemoglobin levels or a higher frequency of anemia have been reported in those with insufficient iodine compared with those with adequate iodine levels (10, 11). However, this biological plausibility does not imply that household salt iodization is a direct causal determinant of childhood anemia; rather, it may reflect contexts of nutritional vulnerability in which multiple deficiencies coexist.

Because natural dietary sources of iodine are limited, universal salt iodization is a low-cost intervention that is widely implemented and key to preventing iodine deficiency disorders (12, 13). Nevertheless, heterogeneity in household coverage of adequately iodized salt has been described, with gaps according to area of residence and wealth level (14). In addition, evidence examining the link between iodine status and childhood anemia remains limited and heterogeneous, and is based mainly on individual biomarkers (9–11). In Peru, although data are available on anemia prevalence and iodized salt coverage (15, 16), population-based analyses integrating both indicators remain scarce. Most previous studies linking iodine status with anemia have relied on individual biomarkers in selected populations, whereas household salt iodization is a programmatic indicator that may capture unequal access to nutrition-related interventions rather than child iodine status itself. Clarifying this distinction is important because it frames household salt iodization as a potential surveillance marker of nutritional vulnerability, not as a direct biological proxy for anemia causation. Moreover, because socioeconomic and territorial inequalities may simultaneously influence exposure to nutritional determinants and the risk of childhood anemia, it is relevant to examine whether these conditions combine and modify the distribution of anemia in the population. Therefore, using ENDES 2014–2023 data, this study aimed to estimate the prevalence of anemia and evaluate disparities by the adequacy of iodine content in household salt among girls and boys aged 6 to 59 months in Peru, as well as to explore variation by area of residence and household wealth level.

## 2 Materials and methods

### 2.1 Study design

We conducted an observational analytical cross-sectional study using publicly available secondary data from Peru’s ENDES. Ten consecutive annual survey rounds corresponding to 2014–2023 were pooled for the primary analysis to maximize sample size and the precision of the estimates while preserving temporal comparability in anemia classification. The 2024 survey round was retained only for a prespecified sensitivity analysis because ENDES 2024 incorporated methodological updates related to hemoglobin adjustment and anemia classification. The manuscript was prepared following the STROBE (Strengthening the Reporting of Observational Studies in Epidemiology) guidelines (17), and the checklist is provided in Supplementary material 1.

### 2.2 Data source

ENDES is a population-based repeated cross-sectional survey conducted annually by Peru’s National Institute of Statistics and Informatics (INEI) with technical assistance from the DHS Program (Demographic and Health Surveys). The survey is nationally representative and representative at the departmental level and by urban and rural area of residence (18). ENDES uses a two-stage, probabilistic, stratified, and independent sampling design at the departmental level. In the first stage, clusters or primary sampling units are selected with probability proportional to size; in the second stage, households within each cluster are selected using probabilistic procedures defined by INEI (18). In selected households, women eligible for the individual questionnaire are interviewed, and their children younger than five years are included in the child health module. ENDES collects household and household-member sociodemographic information, as well as maternal and child health and nutrition data. It includes measurement of household salt iodine content using a rapid test kit (RTK) and capillary hemoglobin measurement in children.

### 2.3 Population and sample

The target population comprised children aged 6 to 59 months living in Peruvian households during 2014–2023 who had an assessment of iodine content in household salt. Children were included if they: (a) were 6 to 59 months old at the time of the survey; (b) lived in households where the salt iodine test was performed using the RTK; (c) had a valid hemoglobin measurement; and (d) had complete information for the survey design variables. Records with missing data on the main exposure, hemoglobin measurement, or survey design variables required for weighted estimation were excluded. No imputation was performed. Missingness in descriptive variables not included in the main adjustment set was retained as an explicit missing/not available category when applicable. The selection flowchart is presented in Supplementary Figure S1.

### 2.4 Variables

The outcome variable was the presence of anemia, defined as a dichotomous variable. Anemia was considered present when altitude-adjusted hemoglobin was lower than 11.0 g/dL, according to WHO criteria for children aged 6 to 59 months (19). Additionally, anemia was classified by severity into three categories: mild (hemoglobin 10.0–10.9 g/dL), moderate (7.0–9.9 g/dL), and severe (<7.0 g/dL). In ENDES, hemoglobin is measured using the HemoCue system (HemoCue AB, Ängelholm, Sweden), a portable photometer that measures hemoglobin concentration in capillary blood obtained by finger prick. Measurements were performed by trained field staff following a standardized protocol. In the present study, we used the altitude-adjusted hemoglobin reported by ENDES, in accordance with the survey’s technical procedures (4, 20).

The main exposure variable was the adequacy of iodine content in household salt, assessed using the RTK, a semiquantitative colorimetric test that estimates salt iodine content based on the intensity of the color reaction produced after adding an indicator solution to a household salt sample. ENDES interviewers recorded the result in four categories according to color intensity: 0 parts per million (ppm, no color change), 7 ppm (faint coloration), 15 ppm (blue or purple coloration), and 30 ppm (dark purple coloration). For programmatic purposes, iodization was dichotomized as adequately iodized salt (≥15 ppm) and inadequately iodized salt (<15 ppm) (21, 22). For the main dichotomous exposure, inadequate iodization corresponded to the RTK categories 0 and 7 ppm, whereas adequate iodization corresponded to 15 and 30 ppm. Because the RTK is a semiquantitative test, this classification should be interpreted as an operational approximation of the iodine content in household salt, not as an exact quantitative measurement. Additionally, the original four RTK categories were retained for descriptive and model-based comparisons across recorded iodine levels. Because RTK results are semiquantitative and depend on color intensity, these categories were interpreted as ordered programmatic categories rather than exact iodine concentrations.

We described child age in months (6–11, 12–23, 24–35, 36–47, and 48–59), sex, area of residence, wealth index quintile, maternal education, chronic undernutrition, defined as a height-for-age Z-score (HAZ) < −2, and survey year. For the main models, adjustment was limited to age and sex. Area of residence and wealth quintile were analyzed as disparity dimensions and as stratification or interaction variables, rather than as primary adjustment covariates.

### 2.5 Statistical analysis

Because ten annual surveys were pooled for the primary analysis, the original sampling weights were divided by 10 so that estimates would represent a weighted average for the 2014–2023 period and to avoid interpreting the combined file as an accumulated population total. For the sensitivity analysis including 2024, the original sampling weights were divided by 11. Pooled estimates were interpreted as period averages rather than as a single population observed at one point in time (23). Cluster and stratum identifiers were made unique by year before specifying the survey design. The scaled single-unit stratum option was used to handle strata with only one primary sampling unit.

The main analytical approach and sensitivity analyses were specified before estimating the final models. Statistical analysis was conducted in five stages. First, a descriptive analysis was performed using unweighted absolute frequencies and weighted percentages with 95% confidence intervals (95% CIs). Second, absolute and relative gaps were estimated; the absolute gap was calculated as the difference in anemia prevalence between children living in households with inadequately versus adequately iodized salt, whereas the relative gap was estimated using the crude prevalence ratio (PR) with 95% CI. In addition, age- and sex-standardized prevalences were estimated using marginal predictions from a logistic model, and standardized absolute gaps were calculated from these estimates. Third, adjusted prevalence ratios (aPRs) were estimated using Poisson regression for a complex survey design with robust variance (24), adjusting for age and sex. Variation across the four recorded RTK categories was explored using category-specific estimates and an ordered-category model. Because RTK results are semiquantitative and depend on color intensity, this analysis was interpreted as an ordinal programmatic pattern across recorded categories rather than as a quantitative linear dose–response. Fourth, additive interaction was evaluated between inadequate iodization and extreme poverty, defined as belonging to wealth quintile 1 versus quintiles 2–5, and between inadequate iodization and rural residence (rural vs. urban). The relative excess risk due to interaction (RERI), attributable proportion due to interaction (AP), and synergy index (S) were calculated (25). Exploratory multiplicative interaction models were fitted as a supplementary complement because interaction estimates may be scale dependent. Fifth, prespecified sensitivity analyses were performed. The first included the 2024 survey round to evaluate the robustness of the primary 2014–2023 analysis. The second excluded 2023 because of anomalies observed in the exposure. The third compared 7 ppm with ≥15 ppm after excluding households with 0 ppm iodine content. The fourth restricted the analysis to children aged 6–23 months to assess the robustness of the association in an age subgroup with high prevalence. All analyses were performed in Stata 17.0 and R 4.3.

### 2.6 Ethical considerations

This study used publicly available anonymized secondary data; therefore, no additional ethical review was required for this analysis. ENDES obtained the original informed consent in accordance with its field procedures. No additional informed consent from the authors was required for the secondary analysis of anonymized data. According to international ethical guidelines, secondary analysis of data without personal identifiers may be considered minimal-risk research and may be exempt from institutional ethical review (26).

## 3 Results

### 3.1 Characteristics of the study sample

The analytical sample included 110,561 children aged 6 to 59 months (Table 1). Most lived in households with adequately iodized salt (≥15 ppm) (*n* = 96,348), whereas 14,213 lived in households with inadequately iodized salt (<15 ppm). Overall, 11.8% of participants were aged 6–11 months, 69.0% were 12–47 months, and 19.2% were 48–59 months. Likewise, 51.0% were boys, and 78.3% lived in urban areas. Regarding socioeconomic status, 63.2% belonged to the poorest to middle wealth quintiles, and 49.2% of mothers had secondary education. Chronic undernutrition was present in 11.0% of children. The average weighted prevalence of anemia was 31.1% (95% CI: 30.7–31.6); the unweighted crude proportion was 34.6%.

**Table 1:** Characteristics of the sample of children aged 6–59 months according to household salt iodization.

Characteristic	Inadequately iodized salt (<15 ppm) *n* (%)	Adequately iodized salt (≥15 ppm) *n* (%)	Total (*N* = 110,561) *n* (%)
Age in months			
6–11 months	1748 (12.1)	11,527 (11.8)	13,275 (11.8)
12–23 months	3,509 (24.9)	23,178 (24.2)	26,687 (24.3)
24–35 months	3,271 (23.2)	22,225 (22.9)	25,496 (23.0)
36–47 months	3,043 (20.9)	20,874 (21.8)	23,917 (21.7)
48–59 months	2,642 (19.0)	18,544 (19.3)	21,186 (19.2)
Sex			
Male	7,337 (51.6)	49,039 (51.0)	56,376 (51.0)
Female	6,876 (48.4)	47,309 (49.0)	54,185 (49.0)
Area of residence			
Urban	8,669 (67.1)	68,314 (79.8)	76,983 (78.3)
Rural	5,544 (32.9)	28,034 (20.2)	33,578 (21.7)
Wealth quintile			
Poorest	5,110 (30.0)	24,626 (17.3)	29,736 (18.8)
Poorer	4,178 (27.0)	25,761 (21.9)	29,939 (22.5)
Middle	2,595 (20.0)	20,203 (22.2)	22,798 (21.9)
Richer	1,512 (13.0)	15,171 (20.8)	16,683 (19.9)
Richest	818 (10.0)	10,587 (17.8)	11,405 (16.9)
Maternal education level			
No education	259 (1.7)	1,202 (1.0)	1,461 (1.1)
Primary	3,445 (23.7)	18,059 (15.5)	21,504 (16.5)
Secondary	7,103 (48.6)	47,229 (49.3)	54,332 (49.2)
Higher	3,354 (26.0)	29,574 (34.1)	32,928 (33.1)
Anemia severity			
No anemia	8,689 (64.3)	63,644 (69.5)	72,333 (68.9)
Mild	3,646 (23.7)	22,584 (21.4)	26,230 (21.7)
Moderate	1840 (11.7)	9,945 (8.9)	11,785 (9.3)
Severe	38 (0.3)	175 (0.2)	213 (0.2)
Chronic undernutrition (HAZ < −2)			
No	11,880 (84.2)	83,480 (89.1)	95,360 (88.5)
Yes	2,266 (15.2)	12,516 (10.4)	14,782 (11.0)
Missing/not available	67 (0.6)	352 (0.4)	419 (0.5)

Peru ENDES, 2014–2023. ppm: parts per million; HAZ: height-for-age Z score. Absolute frequencies are unweighted and percentages are weighted according to the complex survey design. Missing/not available categories are explicitly shown when applicable.

When characteristics were compared by household salt iodization, the distributions by sex and age group were similar between households with adequate and those with inadequate salt. However, the latter had a higher weighted proportion of children living in rural areas (32.9% vs. 20.2%) and belonging to the two lowest wealth quintiles (57.0% vs. 39.2%). They also had a higher proportion of mothers with primary education (23.7% vs. 15.5%) and a higher frequency of chronic child undernutrition (15.2% vs. 10.4%) compared with households with adequate salt.

### 3.2 Prevalence of anemia according to household salt iodization

**Figure 1:**
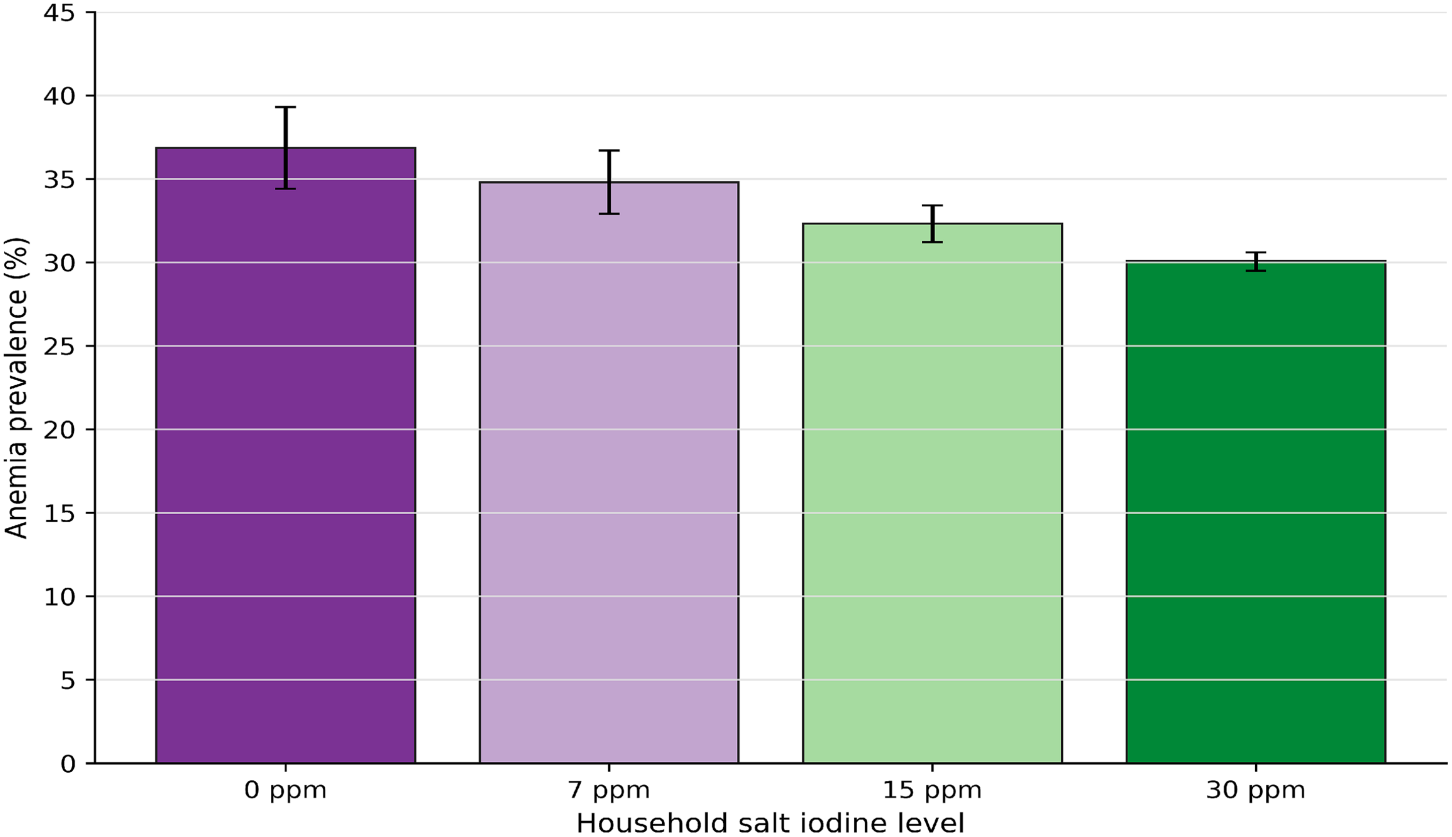
Weighted prevalence of anemia (Hb < 11.0 g/dL) in children aged 6–59 months according to four levels of household salt iodization determined by rapid colorimetric kit: 0 ppm, 7 ppm, 15 ppm, and 30 ppm. Peru ENDES 2014–2023. Error bars represent 95% confidence intervals.

Anemia prevalence was higher among children living in households with inadequately iodized salt than among those living in households with adequately iodized salt (35.73% vs. 30.49%) (Table 2). When iodine content was analyzed in the four RTK categories, anemia prevalence was highest at 0 ppm (36.87%) and lowest at 30 ppm (30.08%) (Figure 1). The ordered-category model was consistent with lower anemia prevalence across higher RTK categories (aPR per one-category higher RTK level = 0.94; 95% CI: 0.92–0.95), but this pattern should be interpreted as descriptive variation across recorded RTK categories rather than as a precise quantitative dose–response gradient.

**Table 2:** Prevalence of anemia according to household salt iodization level, overall and by subgroups.

Dimension	Subgroup/level	Category	*n*	Weighted prevalence of anemia, % (95% CI)
Main comparison	Total	Inadequately iodized salt (<15 ppm)	14,213	35.73 (34.3–37.1)
	Total	Adequately iodized salt (≥15 ppm)	96,348	30.49 (30.0–31.0)
Household salt iodization level	Total	0 ppm	6,124	36.87 (34.4–39.3)
	Total	7 ppm	8,089	34.79 (32.9–36.7)
	Total	15 ppm	19,467	32.32 (31.2–33.4)
	Total	30 ppm	76,881	30.08 (29.5–30.6)
Age group	6–11 months	Inadequately iodized salt (<15 ppm)	1748	61.09 (56.2–66.0)
	6–11 months	Adequately iodized salt (≥15 ppm)	11,527	54.01 (52.4–55.6)
	12–23 months	Inadequately iodized salt (<15 ppm)	3,509	49.44 (46.1–52.7)
	12–23 months	Adequately iodized salt (≥15 ppm)	23,178	43.62 (42.6–44.7)
	24–35 months	Inadequately iodized salt (<15 ppm)	3,271	32.39 (29.0–35.8)
	24–35 months	Adequately iodized salt (≥15 ppm)	22,225	26.21 (25.3–27.1)
	36–47 months	Inadequately iodized salt (<15 ppm)	3,043	24.69 (21.8–27.6)
	36–47 months	Adequately iodized salt (≥15 ppm)	20,874	20.35 (19.4–21.3)
	48–59 months	Inadequately iodized salt (<15 ppm)	2,642	17.75 (15.3–20.2)
	48–59 months	Adequately iodized salt (≥15 ppm)	18,544	16.15 (15.3–17.0)
Sex	Male	Inadequately iodized salt (<15 ppm)	7,337	36.80 (34.7–38.9)
	Male	Adequately iodized salt (≥15 ppm)	49,039	31.47 (30.8–32.2)
	Female	Inadequately iodized salt (<15 ppm)	6,876	34.58 (32.5–36.7)
	Female	Adequately iodized salt (≥15 ppm)	47,309	29.46 (28.8–30.1)
Area of residence	Rural	Inadequately iodized salt (<15 ppm)	5,544	42.38 (40.3–44.5)
	Rural	Adequately iodized salt (≥15 ppm)	28,034	39.36 (38.5–40.2)
	Urban	Inadequately iodized salt (<15 ppm)	8,669	32.47 (30.7–34.3)
	Urban	Adequately iodized salt (≥15 ppm)	68,314	28.24 (27.7–28.8)
Wealth quintile	Poorest	Inadequately iodized salt (<15 ppm)	5,110	43.35 (40.7–46.0)
	Poorest	Adequately iodized salt (≥15 ppm)	24,626	41.11 (40.1–42.1)
	Poorer	Inadequately iodized salt (<15 ppm)	4,178	41.43 (38.4–44.5)
	Poorer	Adequately iodized salt (≥15 ppm)	25,761	35.51 (34.6–36.5)
	Middle	Inadequately iodized salt (<15 ppm)	2,595	32.67 (29.0–36.4)
	Middle	Adequately iodized salt (≥15 ppm)	20,203	30.20 (29.1–31.3)
	Richer	Inadequately iodized salt (<15 ppm)	1,512	24.54 (19.7–29.4)
	Richer	Adequately iodized salt (≥15 ppm)	15,171	25.70 (24.5–26.9)
	Richest	Inadequately iodized salt (<15 ppm)	818	18.06 (11.9–24.2)
	Richest	Adequately iodized salt (≥15 ppm)	10,587	19.97 (18.6–21.4)

Peru ENDES, 2014–2023. Weighted prevalences and 95% confidence intervals (95% CIs) are presented. Anemia was defined as altitude-adjusted hemoglobin <11.0 g/dL. Panel B: prevalences correspond to total anemia at each iodization level. Panels C–F: prevalences are reported as the percentage with anemia within each salt group, stratified by subgroup. Panel B. Household salt iodization level; Panel C. Age group; Panel D. Sex; Panel E. Area of residence; Panel F. Wealth quintile.

Anemia was concentrated in the youngest age groups and decreased with age in both iodization groups; for example, in households with inadequate salt, it reached 61.09% among children aged 6–11 months and decreased to 17.75% among those aged 48–59 months. In both sexes, the prevalence of anemia was higher in households with inadequate salt than in those with adequate salt. Among boys, prevalences were 36.80 and 31.47%, respectively; among girls, 34.58 and 29.46%. These differences were also maintained by area of residence, both in rural areas (42.38% vs. 39.36%) and urban areas (32.47% vs. 28.24%). By wealth quintile, anemia was more frequent in the poorest and poorer quintiles, with higher values in households with inadequate salt (43.35 and 41.43%) than in households with adequate salt (41.11 and 35.51%).

In the analysis restricted to children aged 6–23 months, the same pattern observed in the full sample was maintained, with higher anemia prevalence in households with inadequately iodized salt than in those with adequately iodized salt (53.26% vs. 47.02%), together with lower prevalence across higher recorded RTK categories (Supplementary Table S4).

### 3.3 Absolute gaps and relative measures according to household salt iodization

Anemia prevalence was higher among children living in households with inadequately iodized salt than among those living in households with adequately iodized salt (35.73% vs. 30.49%). The absolute gap was 5.24 percentage points (pp) (95% CI: 3.7–6.7). In relative terms, this difference corresponded to a crude PR of 1.17 (95% CI: 1.13–1.21). After age- and sex-standardization, the absolute gap remained of similar magnitude (4.90 pp.; 95% CI: 3.7–6.1) (Table 3).

**Table 3:** Absolute gaps and relative measures of the association between household salt iodization and anemia in children aged 6–59 months.

Comparison	Prev./Diff. (%)	95% CI	Crude PR	95% CI	aPR	95% CI
Panel A. Dichotomous salt iodization (inadequate vs. adequate)						
Inadequately iodized salt (<15 ppm)	35.73	34.3–37.1	1.17	1.13–1.21	1.16	1.12–1.20
Adequately iodized salt (≥15 ppm)	30.49	30.0–31.0	Ref.	--	Ref.	--
Absolute gap (pp)	5.24	3.7–6.7	*p* < 0.001	--	4.90	3.7–6.1
Panel B. Iodization level (ref.: 30 ppm)						
0 ppm	36.87	34.4–39.3	1.23	1.16–1.29	1.20	1.15–1.27
7 ppm	34.79	32.9–36.7	1.16	1.10–1.21	1.15	1.10–1.20
15 ppm	32.32	31.2–33.4	1.07	1.04–1.11	1.07	1.04–1.11
30 ppm	30.08	29.5–30.6	Ref.	--	Ref.	--
Panel C. Standardized differences and paired PRs between levels						
0 ppm vs. 30 ppm	6.17	4.4–8.0	1.23	1.16–1.29	1.20	1.15–1.27
7 ppm vs. 30 ppm	4.55	3.0–6.0	1.16	1.10–1.21	1.15	1.10–1.20
15 ppm vs. 30 ppm	2.12	1.1–3.2	1.07	1.04–1.11	1.07	1.04–1.11
7 ppm vs. 0 ppm	−1.62	−3.8 to 0.6	0.94	0.88–1.01	0.96	0.90–1.02
15 ppm vs. 0 ppm	−4.05	−6.0 to −2.1	0.88	0.83–0.93	0.89	0.84–0.94
15 ppm vs. 7 ppm	−2.43	−4.1 to −0.7	0.93	0.88–0.98	0.93	0.89–0.98
Panel D. Gaps by subgroup (inadequate vs adequate)						
Rural area	3.02	0.7–5.3	1.08	1.03–1.13	1.08	1.03–1.14
Urban area	4.23	2.3–6.1	1.15	1.10–1.21	1.13	1.08–1.19
Quintile 1 (poorest)	2.23	−0.6 to 5.1	1.05	1.00–1.11	1.06	1.01–1.11
Quintile 2 (poorer)	5.92	2.8–9.1	1.17	1.10–1.24	1.15	1.09–1.22
Quintile 3 (middle)	2.48	−1.4 to 6.3	1.08	0.99–1.18	1.06	0.98–1.15
Quintile 4 (richer)	−1.16	−6.2 to 3.8	0.96	0.84–1.09	0.97	0.85–1.10
Quintile 5 (richest)	−1.91	−8.2 to 4.4	0.90	0.73–1.12	0.92	0.74–1.13

Peru ENDES, 2014–2023. Children aged 6 to 59 months (*N* = 110,561). Reference: adequate salt (≥15 ppm) or 30 ppm according to panel. Absolute gaps are expressed in percentage points (pp). PR: prevalence ratio; aPR: PR adjusted for age group and sex using Poisson regression for complex survey design. Panel A, gap row: the aPR column shows the age- and sex-standardized gap in pp. “--” indicates that the measure does not apply.

In pairwise comparisons across iodization levels, standardized gaps indicated lower anemia prevalence at higher recorded RTK categories. When comparing 0 ppm with 30 ppm, the standardized gap was 6.17 pp. (95% CI: 4.4–8.0), and when comparing 7 ppm with 30 ppm, it was 4.55 pp. (95% CI: 3.0–6.0). Similarly, the comparison between 15 ppm and 30 ppm showed a gap of 2.12 pp. (95% CI: 1.1–3.2). Among the lower levels, 7 ppm showed a lower prevalence than 0 ppm (−1.62 pp.; 95% CI: −3.8 to 0.6), whereas 15 ppm was lower than 0 ppm (−4.05 pp.; 95% CI: −6.0 to −2.1) and lower than 7 ppm (−2.43 pp.; 95% CI: −4.1 to −0.7) (Table 3, panel C).

**Figure 2:**
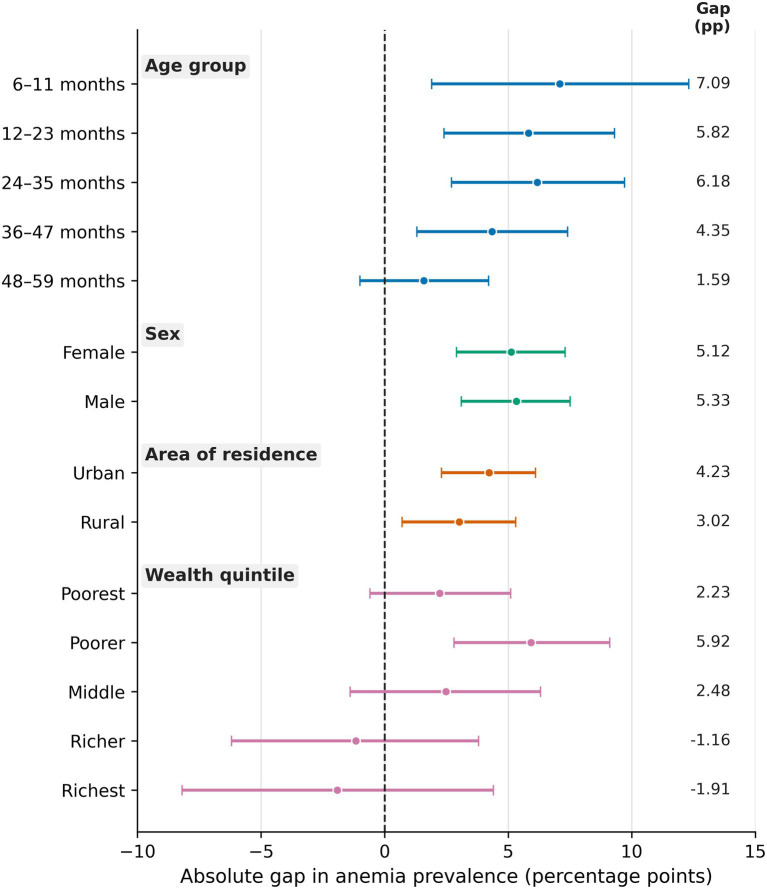
Absolute gaps in anemia prevalence according to inadequate household salt iodization (<15 ppm) versus adequate salt (≥15 ppm), stratified by age group, sex, area of residence, and wealth quintile. Peru ENDES 2014–2023.

In subgroup analysis, the absolute gap between households with inadequate salt and adequate salt was larger in urban areas (4.23 pp.; 95% CI: 2.3–6.1) than in rural areas (3.02 pp.; 95% CI: 0.7–5.3). By wealth quintile, the gap was positive in the first three quintiles, with the largest magnitude in quintile 2 (5.92 pp.; 95% CI: 2.8–9.1), whereas it was negative in quintiles 4 and 5 (Table 3, panel D; Figure 2). In the age- and sex-adjusted relative analysis, households with inadequately iodized salt had a higher prevalence of anemia than households with adequately iodized salt (aPR = 1.16; 95% CI: 1.12–1.20) (Table 3).

Sensitivity analyses showed results consistent with the primary analysis. Including 2024 did not substantially modify the magnitude of the adjusted association (aPR = 1.16; 95% CI: 1.12–1.20), and similar results were observed when 2023 was excluded (aPR = 1.16; 95% CI: 1.12–1.20) (Supplementary Table S3). In the RTK sensitivity analysis excluding households with 0 ppm iodine content, children living in households with 7 ppm had a higher anemia prevalence than those living in households with ≥15 ppm iodine content (34.79% vs. 30.49%; aPR = 1.14; 95% CI: 1.09–1.19), indicating that the main finding was not driven exclusively by households with no detectable iodine in salt (Supplementary Table S6). In the analysis restricted to children aged 6–23 months, the association also remained in the same direction and with a magnitude comparable to that observed in the full sample (aPR = 1.13; 95% CI: 1.09–1.18) (Supplementary Table S4).

### 3.4 Additive interaction between salt iodization and socioeconomic conditions

When extreme poverty was defined as belonging to quintile 1 versus quintiles 2–5, anemia prevalences were 28.27% in households with adequate salt and not in quintile 1, 32.46% in households with inadequate salt and not in quintile 1, 41.11% in households with adequate salt and quintile 1, and 43.35% in households with inadequate salt and quintile 1 (Table 4). The additive-scale indicators did not provide statistical evidence of additive interaction between inadequate iodization and extreme poverty (RERI = −0.058; 95% CI: −0.142 to 0.026; AP = −0.039; S = 0.894).

**Table 4:** Additive-scale interaction between household salt iodization and socioeconomic conditions in relation to childhood anemia.

Type	Combination/measure	Prev./value	95% CI	aPR	95% CI aPR	*p*
Panel A. Salt × extreme poverty (quintile 1 vs quintiles 2–5)						
Combination	Adequately iodized salt + non-Q1 (ref.)	28.27	27.7–28.8	Ref.	--	--
Combination	Inadequately iodized salt + non-Q1	32.46	30.7–34.2	1.14	1.09–1.19	< 0.001
Combination	Adequately iodized salt + Q1	41.11	40.1–42.1	1.41	1.37–1.45	< 0.001
Combination	Inadequately iodized salt + Q1	43.35	40.7–46.0	1.49	1.42–1.56	< 0.001
Interaction	RERI	−0.058	−0.142 to 0.026	--	--	0.173
Interaction	AP	−0.039	−0.097 to 0.018	--	--	0.181
Interaction	S	0.894	0.748 to 1.039	--	--	--
Panel B. Salt × rural residence						
Combination	Adequately iodized salt + urban (ref.)	28.24	27.7–28.8	Ref.	--	--
Combination	Inadequately iodized salt + urban	32.47	30.7–34.3	1.13	1.08–1.19	< 0.001
Combination	Adequately iodized salt + rural	39.36	38.5–40.2	1.36	1.32–1.40	< 0.001
Combination	Inadequately iodized salt + rural	42.38	40.3–44.5	1.48	1.41–1.55	< 0.001
Interaction	RERI	−0.017	−0.103 to 0.069	--	--	0.699
Interaction	AP	−0.011	−0.070 to 0.047	--	--	0.701
Interaction	S	0.966	0.795 to 1.137	--	--	--

Peru ENDES, 2014–2023. aPR adjusted for age and sex using Poisson regression for complex survey design. RERI: relative excess risk due to interaction; AP: attributable proportion due to interaction; S: synergy index. RERI close to 0, AP close to 0, and S close to 1 indicate absence of additive interaction. Interaction estimates should be interpreted cautiously because they are less precise than main-effect estimates. “--” indicates that the measure does not apply.

**Figure 3:**
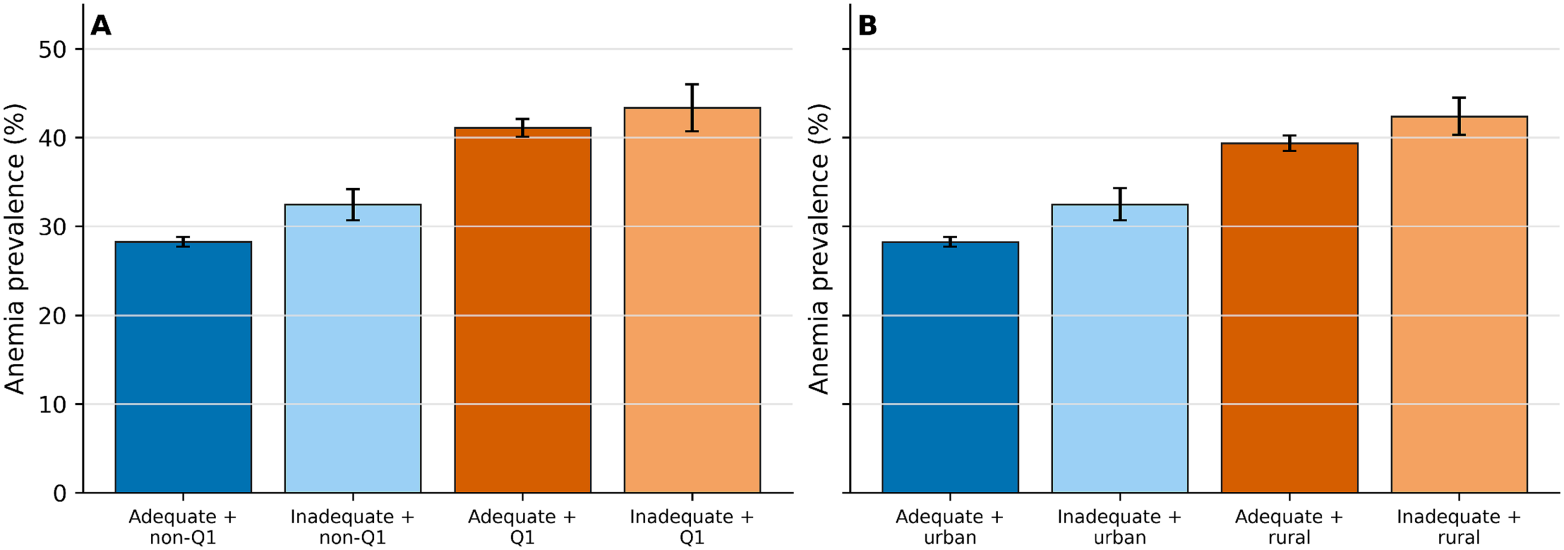
Weighted prevalence of anemia according to combinations of household salt iodization with socioeconomic conditions in children aged 6–59 months, Peru ENDES 2014–2023. Panel A shows combinations of salt iodization with extreme poverty (quintile 1 vs. quintiles 2–5). Panel B shows combinations of salt iodization with area of residence (urban vs. rural). Error bars represent 95% CIs.

Similarly, in the joint analysis of salt iodization and area of residence, anemia prevalence was highest in the rural group with inadequate salt (42.38%), followed by the rural group with adequate salt (39.36%). In contrast, the lowest prevalences were observed in the urban groups with adequate salt (28.24%) and inadequate salt (32.47%) (Table 4). No statistical evidence of additive interaction with rural residence was observed either (RERI = −0.017; 95% CI: −0.103 to 0.069; AP = −0.011; S = 0.966). Because interaction contrasts rely on joint-exposure strata and are generally less precise than main-effect estimates in complex survey data, these findings should be interpreted as absence of statistical evidence for additive-scale interaction rather than definitive evidence that interaction is absent. Exploratory multiplicative interaction analyses are presented in Supplementary Table S7 and did not alter the interpretation based on additive-scale interaction. Figure 3 graphically shows these combinations.

### 3.5 Temporal trends in inadequately iodized salt

**Figure 4:**
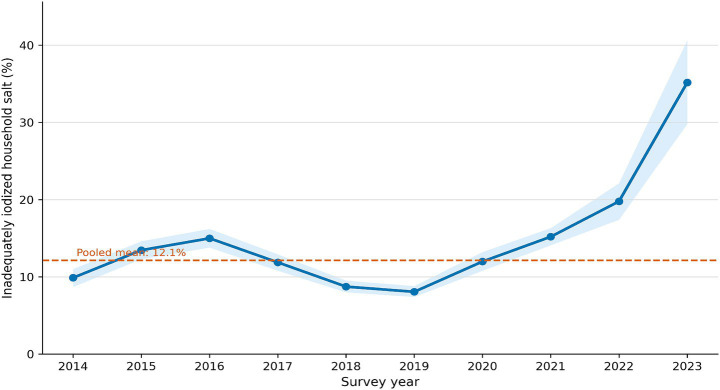
Proportion of households with children aged 6–59 months that use table salt with iodine content below 15 ppm according to rapid colorimetric test (RTK), by survey year. Peru ENDES 2014–2023. The dashed horizontal line indicates the pooled mean (12.1%).

The proportion of households with inadequately iodized salt varied over the 2014–2023 period (Figure 4; Supplementary Table S2). From 2014 to 2019, a progressive decrease was observed, with the lowest value in 2019 (8.06%). From 2020 onward, a progressive increase was observed, reaching a marked peak in 2023 (35.16%). Therefore, the trend should be interpreted as descriptive and potentially influenced by operational, sampling, or measurement changes.

### 3.6 Geographic variability

Anemia prevalence and the proportion of households with inadequately iodized salt showed marked variability across departments (Supplementary Table S5). Anemia prevalence ranged from 22.81% (Tumbes) to 60.15% (San Martín), with also high values in Huánuco (46.59%), Madre de Dios (46.41%), and Callao (45.88%). In parallel, the proportion of inadequately iodized salt was particularly high in San Martín (49.01%), Puno (35.15%), and Moquegua (29.54%), whereas departments such as Pasco (3.52%) and Arequipa (3.80%) showed low proportions (Supplementary Table S5; Supplementary Figure S3).

At the departmental level, an ecological correlation was observed between the proportion of inadequately iodized salt and the prevalence of anemia (Supplementary Figure S2). When the absolute gap in anemia was examined, defined as the prevalence in households with inadequate salt minus the prevalence in households with adequate salt, wider positive gaps were identified in Apurímac (6.6 pp), Junín (4.4 pp), Callao (4.4 pp), Lambayeque (3.9 pp), and Cajamarca (3.9 pp). In contrast, some departments showed gaps close to zero or negative, such as Moquegua (0.04 pp), Lima (−4.6 pp), Huánuco (−4.3 pp), La Libertad (−4.3 pp), and Áncash (−4.2 pp) (Supplementary Table S5).

## 4 Discussion

### 4.1 Main findings

In this analysis of a nationally representative population-based survey, children living in households with inadequately iodized salt had a higher prevalence of anemia than those living in households with adequately iodized salt. The absolute difference was close to five percentage points, and the crude prevalence ratio was approximately 17% higher; after adjustment for age and sex, the relative prevalence remained about 16% higher. These differences persisted after age- and sex-standardization. When iodine content was analyzed across the recorded RTK categories, anemia prevalence was highest at 0 ppm and lowest at 30 ppm, but this pattern should be interpreted as an ordinal programmatic pattern rather than as a precise quantitative dose–response relationship.

In analyses stratified by area of residence and wealth quintiles, gaps were larger in urban areas and tended to concentrate in the lower wealth quintiles. However, the pattern was not strictly monotonic. In particular, the gap was larger in quintile 2 than in quintile 1, whereas in higher wealth quintiles the differences were small or negative. Although the highest prevalences were observed in groups with greater accumulated disadvantage—inadequately iodized salt together with extreme poverty or rural residence—the additive-scale interaction indicators did not provide statistical evidence of excess beyond the sum of individual effects. These findings remained consistent when 2024 was included as a sensitivity analysis, when 2023 was excluded, when the comparison was restricted to 7 ppm versus ≥15 ppm, and in the analysis restricted to children aged 6–23 months, reinforcing the robustness of the results.

### 4.2 Comparison with other studies

The higher prevalence of anemia among children living in households with inadequately iodized salt is consistent with the findings of Khatiwada et al. (9), who found that among Nepalese schoolchildren, iodine insufficiency was associated with lower hemoglobin concentrations and a higher frequency of anemia. Similarly, Simpong et al. (11) reported that, among pregnant women in rural settings, iodine insufficiency was more frequent in women with anemia. Likewise, Farha and Urooj (10) found that among pregnant women in the first trimester, hemoglobin levels differed according to iodine status, as assessed by urinary iodine concentration. Although these studies differ in population and in exposure measurement, as they use individual biomarkers such as urinary iodine, taken together, they suggest that lower iodine status may coexist with a greater burden of anemia in contexts of nutritional vulnerability.

However, our results should be interpreted primarily from a population-disparities perspective rather than as evidence of a specific effect attributable to iodine. Childhood anemia has a multifactorial etiology and reflects the concurrence of nutritional, infectious, environmental, and social determinants (5). Within this framework, inadequate household salt iodization may be understood as a programmatic marker of contexts with greater nutritional vulnerability rather than as a direct causal indicator of anemia. This interpretation is consistent with the frequent coexistence of multiple nutritional deficiencies in early childhood (27).

The pattern observed across the four recorded RTK categories adds descriptive coherence to the main finding, but it should not be interpreted as a causal dose–response relationship. Because exposure was measured using a semiquantitative rapid test kit applied to household salt, and because color categories may be difficult to discriminate precisely, particularly at higher iodine concentrations, this pattern should be understood as an ordered programmatic pattern consistent with an unequal distribution of nutritional and programmatic conditions rather than as a quantitative iodine gradient (21, 22).

From an equity perspective, one of the most relevant findings was the heterogeneity of the gaps according to area of residence and wealth level. The positive differences in the lower wealth quintiles suggest that the greater burden of anemia is concentrated in socially disadvantaged groups, where multiple adverse determinants are likely to converge, a pattern consistent with previous studies documenting socioeconomic inequalities in childhood anemia in Peru (15). This pattern is also consistent with evidence showing that coverage of adequately iodized salt tends to be higher in urban areas and in households of higher socioeconomic status. However, gaps persist within each context (14), and studies conducted in Peru document a socially stratified distribution of salt consumption with inadequate iodine concentration (16). The fact that, in higher wealth quintiles, the gaps were small or even reversed reinforces the idea that disparities in anemia by household salt iodization are not uniform across the population and that, in more advantaged strata, other determinants may carry greater relative weight. Although prevalences were higher in groups with accumulated disadvantage, the additive-scale interaction indicators did not provide statistical evidence of a supra-additive joint effect with extreme poverty or rural residence. This suggests that the greater burden of anemia observed in these groups may be explained by the coexistence and accumulation of conditions of social and nutritional vulnerability rather than by an interaction between the factors evaluated (25).

### 4.3 Public health implications

These findings have implications for equity-oriented nutritional surveillance. The higher prevalence of anemia in households with inadequately iodized salt suggests that this indicator, available in population surveys, may provide complementary information to identify subgroups and territories where the burden of anemia is greater. However, its usefulness should be understood at the descriptive and programmatic levels, not as a substitute for individual biomarkers or as evidence of causality.

The heterogeneity observed according to wealth, residence, and territory also highlights the importance of systematically reporting gaps beyond national averages. In particular, the finding of larger gaps in urban settings suggests that inequality surveillance should not focus exclusively on rural areas. Likewise, differences across wealth quintiles show that although the burden is greater in socially vulnerable groups, anemia is not restricted to extreme poverty.

Temporal and geographic variability in the proportion of households with inadequately iodized salt also reinforces the need to strengthen monitoring of fortification quality throughout the production, distribution, and commercialization chain (28). From a programmatic perspective, these results support integration between iodized salt monitoring, anemia surveillance, and monitoring of nutritional inequalities, especially at the subnational level.

### 4.4 Study limitations

This study has some limitations. First, its cross-sectional design prevents the establishment of temporality between the exposure and the outcome and therefore does not allow causal inference. Second, exposure was assessed using rapid test kits applied to household salt. Although useful for programmatic monitoring, these do not provide a direct measure of the child’s iodine status or an exact quantification of salt iodine content. Quantitative methods, such as titration, are more appropriate for estimating specific iodine concentrations, and exposure misclassification may exist (21, 22). In addition, ENDES measures iodine content in salt available at the household and cannot distinguish inadequate iodization at production from iodine losses during transport, commercialization, household storage, or use. Because iodine retention may vary by packaging, humidity, temperature, storage duration, and handling practices, exposure misclassification may also vary geographically (29). Third, childhood anemia has a multifactorial etiology, and the information available in ENDES does not allow identification of its specific causes, so residual confounding cannot be ruled out (5). Fourth, to preserve temporal comparability, the primary analysis excluded 2024 because of methodological changes in anemia classification and altitude adjustment; nevertheless, including 2024 in the sensitivity analysis did not substantially modify the findings (19, 30). Finally, interaction analyses may have had limited precision because they were based on joint categories of salt iodization with rural residence or extreme poverty; therefore, null interaction findings should be interpreted cautiously. Some subgroup- or department-level estimates may also be less precise due to limited sample sizes in certain strata.

## 5 Conclusion

In this population-based sample of Peruvian children aged 6 to 59 months, the prevalence of anemia was higher among those living in households with inadequately iodized salt. Gaps were heterogeneous across areas of residence and wealth quintiles. Based on the evaluated definition of extreme poverty, the analyses did not provide statistical evidence of additive-scale interaction with extreme poverty or rural residence. Taken together, the findings support an interpretation centered on population disparities and suggest that household salt iodization may function as a programmatic marker of nutritional vulnerability. However, its usefulness for surveillance and potential targeting of actions requires further validation through studies incorporating individual biomarkers and more robust analytical designs.

## Data Availability

Publicly available datasets were analyzed in this study. This data can be found here: https://proyectos.inei.gob.pe/microdatos/.
