# Supplementary material for: Childhood anemia and household salt iodization in Peru: population disparities among children aged 6–59 months
Source: Front Nutr. 2026 Jun 12;13:1865961. doi: 10.3389/fnut.2026.1865961 (PMC13307304; doi:10.3389/fnut.2026.1865961)

**Supplementary Material**

**Supplementary Text S1. STROBE Checklist**

| **No.** | **STROBE recommendation** | **Location in manuscript** |
| --- | --- | --- |
| 1a | Indicate the study design in the title or abstract using a commonly used term. | Title: “cross-sectional analysis of ENDES 2014-2023”. |
| 1b | Provide in the abstract an informative and balanced summary of what was done and what was found. | Abstract: includes background, objective, cross-sectional design, ENDES 2014-2023, population aged 6–59 months, exposure, outcome, main methods, key results, and a cautious conclusion. |
| 2 | Background/rationale: explain the reasons and scientific background for the investigation. | Introduction, paragraphs 1–3: global, regional, and national burden of anemia; multifactorial etiology; relevance of iodine; universal salt iodization; heterogeneity in coverage; gap in population-based evidence in Peru. |
| 3 | Objectives: state the specific objectives, including any prespecified hypotheses. | Introduction, final paragraph: estimate anemia prevalence and evaluate disparities according to the adequacy of iodine content in household salt, including variation by area and wealth. |
| 4 | Study design: present key elements of study design early in the paper. | Methods, “Study design”: observational analytical cross-sectional study using secondary ENDES 2014-2023 data. |
| 5 | Setting: describe the setting, locations, and relevant dates. | Methods, “Study design” and “Data source”: Peru ENDES, annual surveys 2014-2023, national, departmental, and area-of-residence representativeness. |
| 6 | Participants: give the eligibility criteria, and the sources and methods of selection. | Methods, “Population and sample”: inclusion of children aged 6–59 months with valid RTK, valid hemoglobin, and complete information for survey design; exclusion of relevant missing data. |
| 7 | Variables: clearly define outcomes, exposures, predictors, potential confounders, and effect modifiers. | Methods, “Variables”: anemia as outcome; salt iodization as main exposure; ordinal iodine levels; age, sex, area, wealth, maternal education, chronic undernutrition, and year; interaction with extreme poverty and rural residence. |
| 8 | Data sources/measurement: give sources of data and details of methods of assessment. | Methods, “Data source” and “Variables”: hemoglobin measured with HemoCue; altitude-adjusted hemoglobin reported by ENDES; RTK for salt iodine; sociodemographic variables obtained from ENDES. |
| 9 | Bias: describe any efforts to address potential sources of bias. | Methods, "Statistical analysis": use of weights, complex design, unique identifiers by year, age- and sex-standardization, sensitivity analyses including 2024, excluding 2023, RTK threshold analysis, multiplicative interaction analysis, and analysis restricted to ages 6-23 months. Discussion: exposure misclassification, residual confounding, and non-causality. |
| 10 | Study size: explain how the study size was arrived at. | Methods, “Population and sample”: sample derived from availability of eligible children in ENDES 2014-2023. Results: final analytical sample of 110,561 children. |
| 11 | Quantitative variables: explain how quantitative variables were handled in the analyses. | Methods, “Variables”: age in categories; wealth in quintiles; iodine in 0, 7, 15, and 30 ppm categories, plus dichotomization as <15/≥15 ppm. |
| 12a | Statistical methods: describe all statistical methods, including those used to control for confounding. | Methods, “Statistical analysis”: unweighted frequencies and weighted percentages; absolute and relative gaps; age- and sex-standardized prevalences; crude PRs and aPRs using Poisson regression; main adjustment for age and sex. |
| 12b | Describe any methods used to examine subgroups and interactions. | Methods, “Statistical analysis”: subgroup analyses according to residence and wealth quintile; additive interaction using RERI, AP, and S for extreme poverty and rural residence. |
| 12c | Explain how missing data were addressed. | Methods, “Population and sample”: exclusion of records with missing data in RTK or survey design variables. Table 1: missing/not available categories for maternal education and HAZ. |
| 12d | If applicable, explain how case–control matching was addressed. | Not applicable (cross-sectional study). |
| 12e | Describe any sensitivity analyses. | Methods, "Statistical analysis": inclusion of 2024, exclusion of 2023, RTK threshold sensitivity analysis, multiplicative interaction analysis, and analysis restricted to children aged 6-23 months. |
| 13a | Report the number of individuals at each stage of the study. | Results, “Characteristics of the study sample” and Figure S1: final analytical sample of 110,561 children. |
| 13b | Give reasons for non-participation at each stage. | Figure S1: exclusions for age outside the range, invalid hemoglobin, invalid RTK, and missing survey design variables. |
| 13c | Consider use of a flow diagram. | Figure S1. |
| 14a | Descriptive data: characteristics of study participants and information on exposures and potential confounders. | Results and Table 1: age, sex, residence, wealth, maternal education, anemia severity, chronic undernutrition, and distribution according to iodization. |
| 14b | Indicate number of participants with missing data for each variable of interest. | Table 1: missing/not available categories are reported for variables with incomplete data. |
| 15 | Outcome data: report numbers of outcome events or summary measures. | Results and Table 1: overall weighted anemia prevalence of 31.1% (95% CI: 30.9–31.7); unweighted crude proportion of 34.7%. |
| 16a | Main results: give unadjusted and adjusted estimates with 95% CIs. | Results and Table 3: prevalence 35.95% vs 30.64%; absolute gap 5.31 pp; crude PR 1.17; standardized gap 4.99 pp; aPR 1.16. |
| 16b | If continuous variables were categorized, report category boundaries. | Methods and Tables 1–2: age, wealth, and iodine levels. |
| 16c | If relevant, consider translating estimates of relative risk into absolute risk. | Table 3: PRs and absolute gaps in percentage points are reported simultaneously. |
| 17 | Other analyses: report analyses of subgroups, interactions, and sensitivity. | Results: subgroups (Table 2, Table 3), additive interaction (Table 4), temporal sensitivity and inclusion of 2024 (Table S3), RTK threshold sensitivity (Table S6), multiplicative interaction (Table S7), analysis of ages 6-23 months (Table S4), temporal trends (Figure 1, Table S2), and geographic variability (Table S5, Figures S2 and S3). |
| 18 | Key results: summarize key results with reference to study objectives. | Discussion, “Main findings”: higher prevalence of anemia in households with inadequately iodized salt, heterogeneity by wealth and residence, absence of supra-additive interaction under extreme poverty, and consistency across sensitivity analyses. |
| 19 | Limitations: discuss limitations of the study, taking into account sources of potential bias or imprecision. | Discussion, "Study limitations": cross-sectional design, RTK not an individual biomarker nor exact quantification, possible misclassification from production, transport, storage, and handling, multifactorial etiology, residual confounding, 2024 comparability handled by sensitivity analysis, and lower precision in some subgroups and interaction analyses. |
| 20 | Interpretation: give a cautious overall interpretation considering objectives, limitations, multiplicity of analyses, results from similar studies, and other relevant evidence. | Discussion: interpretation centered on disparities and nutritional vulnerability, not on specific iodine causality. |
| 21 | Generalisability: discuss the generalisability (external validity) of the study results. | Discussion/Limitations: ENDES has national, departmental, and area-level representativeness; findings are generalizable to Peruvian children aged 6–59 months living in households assessed by ENDES. |
| 22 | Funding: give the source of funding and the role of the funders. | Declarations: study funded by the Vice-Rectory for Research; no sponsor. |

STROBE: Strengthening the Reporting of Observational Studies in Epidemiology.

**Table S2. Proportion of households with inadequately iodized salt by survey year. Peru ENDES, 2014-2023.**

| **Year** | **Inadequately iodized salt (<15 ppm) %** | **95% CI** | **Adequately iodized salt (>=15 ppm) %** |
| --- | --- | --- | --- |
| 2014 | 9.88 | 8.7-11.0 | 90.12 |
| 2015 | 13.45 | 12.3-14.6 | 86.55 |
| 2016 | 14.99 | 13.8-16.2 | 85.01 |
| 2017 | 11.87 | 10.8-12.9 | 88.13 |
| 2018 | 8.73 | 8.0-9.5 | 91.27 |
| 2019 | 8.06 | 7.4-8.8 | 91.94 |
| 2020 | 11.98 | 10.8-13.2 | 88.02 |
| 2021 | 15.19 | 14.1-16.3 | 84.81 |
| 2022 | 19.77 | 17.4-22.1 | 80.23 |
| 2023 | 35.16 | 29.8-40.6 | 64.84 |
| Pooled | 12.14 | 11.8-12.5 | 87.86 |

Weighted proportions according to the ENDES complex survey design. ppm: parts per million. Values are presented for the primary 2014-2023 analysis; 2024 was evaluated as a sensitivity analysis rather than included in the primary temporal trend.

**Table S3. Age- and sex-adjusted prevalence ratios for anemia according to household salt iodization: main analysis and sensitivity analyses. Peru ENDES, 2014-2023.**

| **Analysis** | **Variable** | **Category** | **PR** | **95% CI** | **p** |
| --- | --- | --- | --- | --- | --- |
| Main 2014–2023 | Adequately iodized salt ≥15 ppm (reference) | Reference | 1.000 |  |  |
| Main 2014–2023 | Salt iodization | Inadequately iodized salt <15 ppm | 1.16 | 1.122-1.200 | <0.001 |
| Main 2014–2023 | Age (months) | 12–23 months | 0.808 | 0.783-0.834 | <0.001 |
| Main 2014–2023 | Age (months) | 24–35 months | 0.491 | 0.473-0.511 | <0.001 |
| Main 2014–2023 | Age (months) | 36–47 months | 0.38 | 0.364-0.398 | <0.001 |
| Main 2014–2023 | Age (months) | 48–59 months | 0.298 | 0.283-0.314 | <0.001 |
| Main 2014–2023 | Sex | Male | 1.069 | 1.042-1.096 | <0.001 |
| Sensitivity including 2024 | Adequately iodized salt ≥15 ppm (reference) | Reference | 1.000 |  |  |
| Sensitivity including 2024 | Salt iodization | Inadequately iodized salt <15 ppm | 1.162 | 1.124-1.202 | <0.001 |
| Sensitivity including 2024 | Age (months) | 12–23 months | 0.803 | 0.779-0.828 | <0.001 |
| Sensitivity including 2024 | Age (months) | 24–35 months | 0.488 | 0.470-0.507 | <0.001 |
| Sensitivity including 2024 | Age (months) | 36–47 months | 0.378 | 0.362-0.395 | <0.001 |
| Sensitivity including 2024 | Age (months) | 48–59 months | 0.299 | 0.284-0.315 | <0.001 |
| Sensitivity including 2024 | Sex | Male | 1.071 | 1.044-1.097 | <0.001 |
| Sensitivity excluding 2023 | Adequately iodized salt ≥15 ppm (reference) | Reference | 1.000 |  |  |
| Sensitivity excluding 2023 | Salt iodization | Inadequately iodized salt <15 ppm | 1.16 | 1.121-1.200 | <0.001 |
| Sensitivity excluding 2023 | Age (months) | 12–23 months | 0.808 | 0.783-0.834 | <0.001 |
| Sensitivity excluding 2023 | Age (months) | 24–35 months | 0.491 | 0.473-0.511 | <0.001 |
| Sensitivity excluding 2023 | Age (months) | 36–47 months | 0.381 | 0.364-0.398 | <0.001 |
| Sensitivity excluding 2023 | Age (months) | 48–59 months | 0.298 | 0.282-0.314 | <0.001 |
| Sensitivity excluding 2023 | Sex | Male | 1.069 | 1.042-1.096 | <0.001 |

PR: prevalence ratio; 95% CI: 95% confidence interval. Models estimated using weighted Poisson regression for complex survey design, adjusted for age groups and sex. Reference categories: adequate salt (>=15 ppm), age 6-11 months, and female sex. Sensitivity analyses included ENDES 2024 and excluded 2023.

**Table S4. Analysis restricted to the subgroup of children aged 6-23 months. Peru ENDES, 2014-2023.**

| **Measure** | **Estimate** | **95% CI** | **p** |
| --- | --- | --- | --- |
| Total N (unweighted) | 39 962 |  |  |
| N with inadequate salt | 5 257 |  |  |
| N with adequate salt | 34 705 |  |  |
| Overall anemia prevalence | 47.80% | 47.0-48.6 |  |
| Inadequately iodized salt (<15 ppm) | 53.26% | 50.6-55.9 |  |
| Adequately iodized salt (≥15 ppm) | 47.02% | 46.1-47.9 |  |
| 0 ppm | 54.16% | 50.1-58.3 |  |
| 7 ppm | 52.51% | 48.9-56.1 |  |
| 15 ppm | 48.43% | 46.3-50.6 |  |
| 30 ppm | 46.71% | 45.7-47.7 |  |
| Crude PR, inadequate vs adequate | 1.133 | 1.088-1.180 | <0.001 |
| PR adjusted for age/sex | 1.132 | 1.087-1.179 | <0.001 |

Subgroup: children aged 6 to 23 months (unweighted N = 39,962). PR adjusted for age group and sex. Reference: adequate salt (>=15 ppm). '--' indicates that the measure does not apply.

**Table S5. Anemia prevalence, proportion of households with inadequately iodized salt, and absolute gap in anemia by department. Peru ENDES, 2014-2023.**

| **Department** | **Anemia prevalence (%)** | **Inadequately iodized salt (%)** | **Gap (pp)** |
| --- | --- | --- | --- |
| Amazonas | 31.75 | 16.61 | 3.2 |
| Áncash | 31.20 | 11.10 | -4.2 |
| Apurímac | 39.99 | 9.22 | 6.6 |
| Arequipa | 28.03 | 3.80 | 2.5 |
| Ayacucho | 36.74 | 6.74 | 1.4 |
| Cajamarca | 25.24 | 15.50 | 3.9 |
| Cusco | 26.83 | 5.26 | 2.6 |
| Huancavelica | 42.57 | 24.77 | 3.9 |
| Huánuco | 46.59 | 7.14 | -4.3 |
| Ica | 31.75 | 8.95 | 1.5 |
| Junín | 30.02 | 5.82 | 4.4 |
| La Libertad | 40.56 | 5.24 | -4.3 |
| Lambayeque | 26.30 | 19.07 | 3.9 |
| Lima | 26.13 | 6.22 | -4.6 |
| Loreto | 24.92 | 5.43 | -2.0 |
| Madre de Dios | 46.41 | 11.78 | 1.8 |
| Moquegua | 44.45 | 29.54 | 0.0 |
| Pasco | 24.52 | 3.52 | 2.8 |
| Piura | 43.96 | 11.37 | 2.5 |
| Puno | 30.17 | 35.15 | 1.1 |
| San Martín | 60.15 | 49.01 | 1.8 |
| Tacna | 34.05 | 10.89 | -3.7 |
| Tumbes | 22.81 | 5.72 | -4.2 |
| Ucayali | 36.48 | 25.94 | 1.0 |
| Callao | 45.88 | 5.39 | 4.4 |

pp: percentage points. Gap = anemia prevalence in households with inadequate salt minus prevalence in households with adequate salt. Negative values indicate that anemia prevalence was higher in households with adequate salt.

**Table S6. Sensitivity analyses using alternative RTK category comparisons. Peru ENDES, 2014-2023.**

| **Comparison** | **N** | **Exposed anemia % (95% CI)** | **Reference anemia % (95% CI)** | **aPR age/sex** | **95% CI** | **p** |
| --- | --- | --- | --- | --- | --- | --- |
| <15 ppm vs >=15 ppm | 110 561 | 35.73 (34.3-37.1) | 30.49 (30.0-31.0) | 1.160 | 1.122-1.200 | <0.001 |
| 7 ppm vs >=15 ppm (0 ppm excluded) | 104 437 | 34.79 (32.9-36.7) | 30.49 (30.0-31.0) | 1.136 | 1.088-1.186 | <0.001 |
| 0 ppm vs >=15 ppm | 102 472 | 36.87 (34.4-39.3) | 30.49 (30.0-31.0) | 1.190 | 1.133-1.249 | <0.001 |
| 0 ppm vs 7 ppm | 14 213 | 36.87 (34.4-39.3) | 34.79 (32.9-36.7) | 1.047 | 0.983-1.114 | 0.151 |
| 15 ppm vs 30 ppm | 96 348 | 32.32 (31.2-33.4) | 30.08 (29.5-30.6) | 1.071 | 1.035-1.107 | <0.001 |

RTK: rapid test kit; aPR: prevalence ratio adjusted for age group and sex. These analyses assess whether the main association depends exclusively on households with no detectable iodine in salt.

**Table S7. Exploratory multiplicative interaction between inadequate household salt iodization and socioeconomic conditions. Peru ENDES, 2014-2023.**

| **Modifier** | **Term** | **Ratio of PRs** | **95% CI** | **p** |
| --- | --- | --- | --- | --- |
| Extreme poverty Q1 vs Q2-Q5 | inadequate salt x modifier | 0.928 | 0.871-0.989 | 0.022 |
| Rural residence | inadequate salt x modifier | 0.958 | 0.897-1.023 | 0.198 |
| Broad poverty Q1-Q2 vs Q3-Q5 | inadequate salt x modifier | 1.064 | 0.986-1.149 | 0.110 |

PR: prevalence ratio. Models were adjusted for age group and sex and are presented as exploratory complements to the additive-scale interaction analysis.

**Table S8. Ordered RTK category model for anemia across recorded salt iodine levels. Peru ENDES, 2014-2023.**

| **Analysis** | **aPR per one-category higher RTK level** | **95% CI** | **p** |
| --- | --- | --- | --- |
| Ordered RTK category model (1 = 0 ppm, 2 = 7 ppm, 3 = 15 ppm, 4 = 30 ppm) | 0.937 | 0.924-0.950 | <0.001 |

aPR: prevalence ratio adjusted for age and sex. This model treats recorded RTK categories as ordered programmatic categories and not as exact quantitative iodine concentrations.

**Supplementary figures**

**Supplementary Figure S1. Flowchart of primary analytic sample definition.**


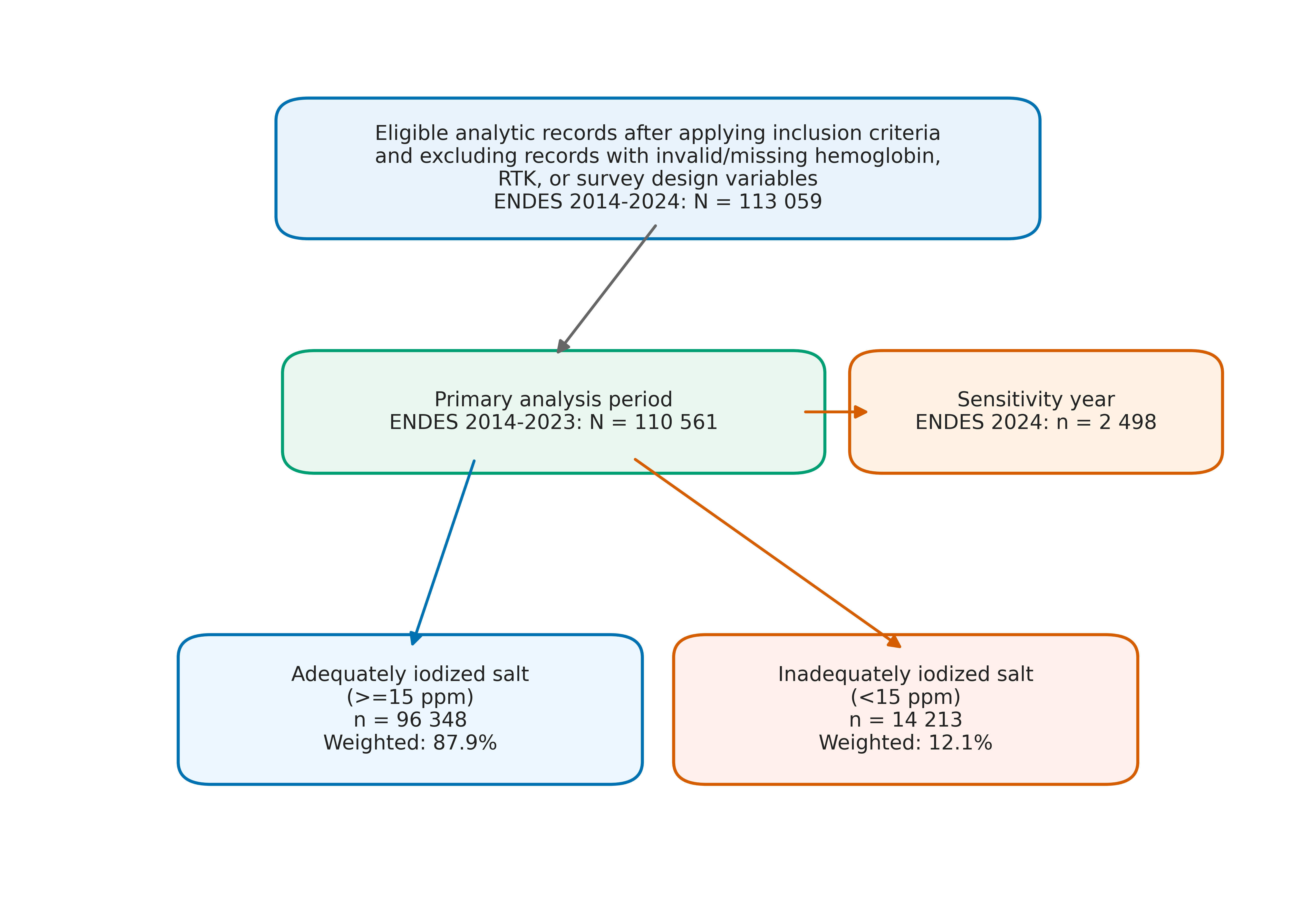


**Supplementary Figure S2. Department-level ecological correlation.**

Scatterplot of the ecological correlation between the proportion of inadequately iodized salt (<15 ppm) and the prevalence of anemia in children aged 6-59 months, by department. Peru ENDES 2014-2023. Each point represents a department with pooled weighted estimates for 2014-2023. The dashed line represents the linear regression.


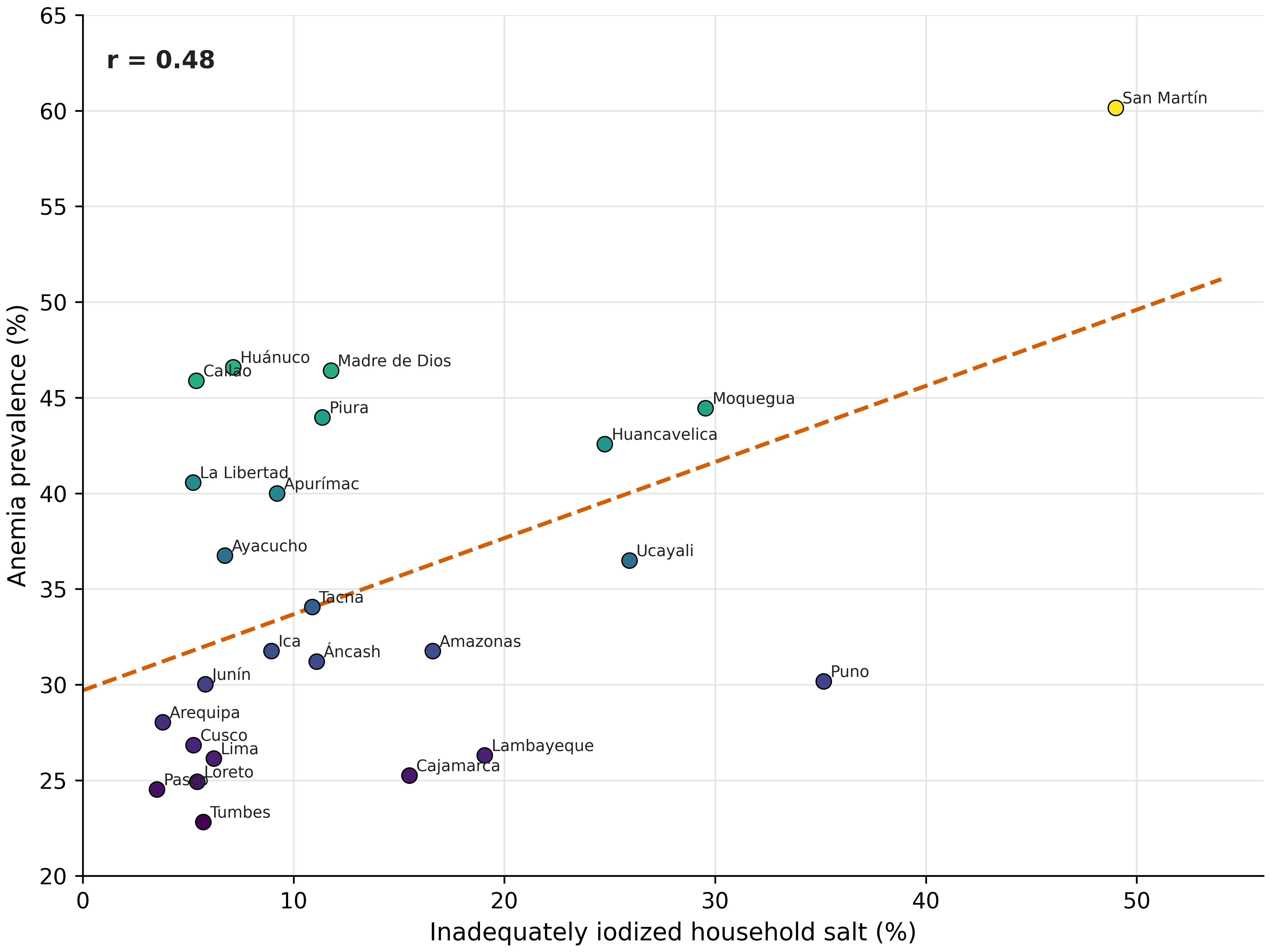


**Supplementary Figure S3. Department-level caterpillar plot.**

Anemia prevalence (Hb <11.0 g/dL) by department in children aged 6-59 months, ordered from lowest to highest. Data from Peru ENDES 2014-2023. The dashed vertical line represents the national average prevalence for the primary period (31.1%).


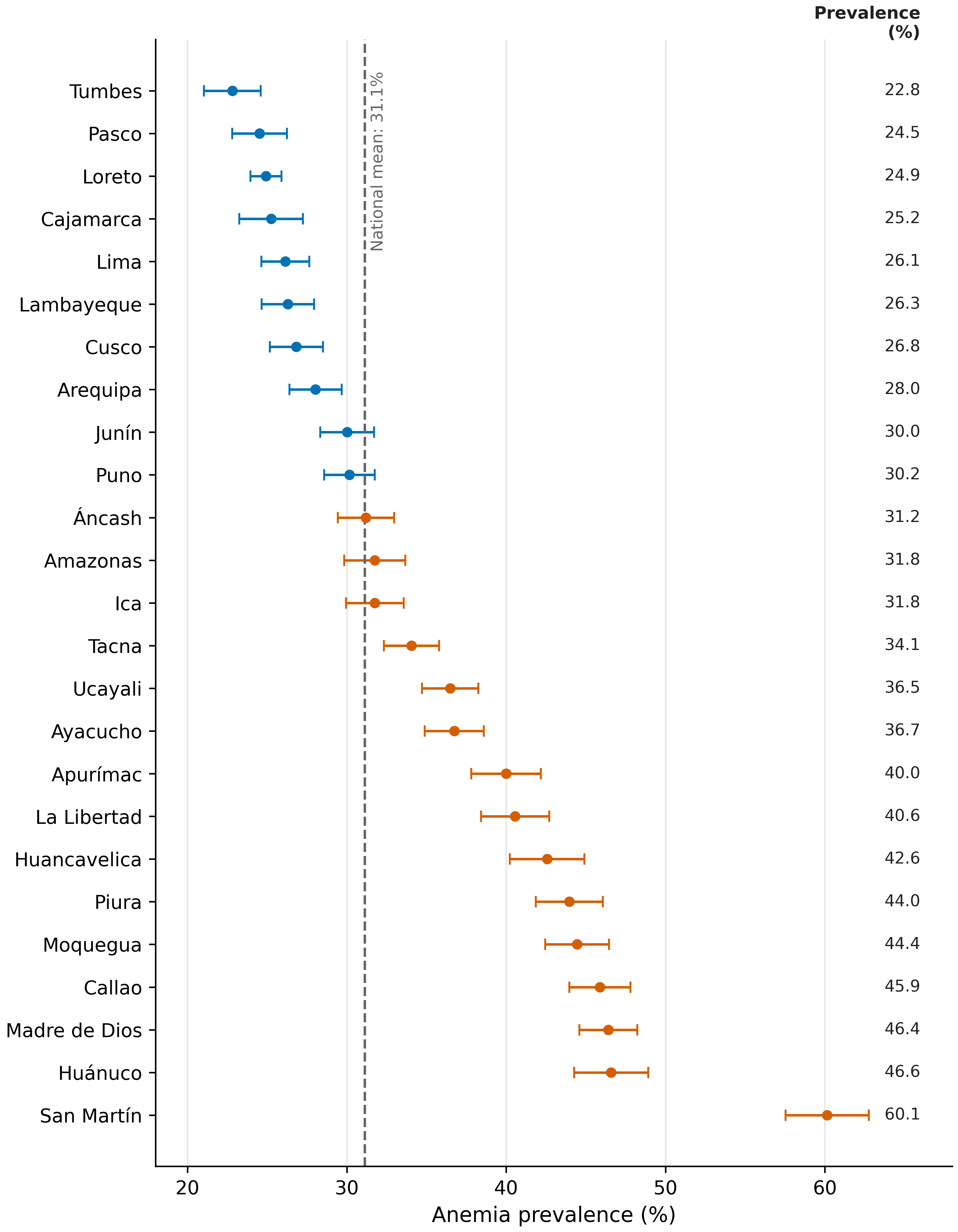

Supplement: Supplementary file 2 [file Table_1.docx]
